# Supplementary material for: Hybrid Models and Biological Model Reduction with PyDSTool
Source: PLoS Comput Biol. 2012 Aug 9;8(8):e1002628. doi: 10.1371/journal.pcbi.1002628 (PMC3415397; doi:10.1371/journal.pcbi.1002628)
Supplement: Text S4 — Complete source code for the PyDSTool package (version 0.88.120504). Includes API documentation and help files linking to web pages. This file is identical to the current public release on Sourceforge.net. (ZIP) [file pcbi.1002628.s004.zip › PyDSTool/html/PyDSTool.Toolbox.DSSRT_tools.DSSRT_info-class.html]

xml version="1.0" encoding="ascii"?


PyDSTool.Toolbox.DSSRT\_tools.DSSRT\_info


| Home | Trees | Indices | Help | PyDSTool |
| --- | --- | --- | --- | --- |

Package PyDSTool ::
Package Toolbox ::
Module DSSRT\_tools ::
Class DSSRT\_info

[hide private]

[frames] | no frames]

# Class DSSRT\_info

source code

```
object --+
         |
        DSSRT_info
```

---


|  |  |
| --- | --- |
| Instance Methods | [hide private] |

|  |  |
| --- | --- |
| \_\_init\_\_(self, name=`'``'`, infty\_val=10000000000.0)  The optional 'infty\_val' argument sets the substitute default finite value used to represent 'infinity', for variables that are not given explicit bounds. | source code |

|  |  |
| --- | --- |
| setUnitBounds(self, coords) | source code |

|  |  |
| --- | --- |
| prepInputs(self, dependencies, nameMap=None) | source code |

|  |  |
| --- | --- |
| prepGraph(self, ics=None, vbars\_int=`{``}`, vars=None)  Call this multiple times to re-randomize the initial vertex selection. | source code |

|  |  |
| --- | --- |
| makeDefaultGraphics(self) | source code |

|  |  |
| --- | --- |
| makeDSSRTcfg(self, model, gen\_name, cfg\_filename)  Make DSSRT configuration file from a PyDSTool Generator object that is embedded in a Model object. | source code |

|  |  |
| --- | --- |
| prepCFG(self)  Prepare .cfg file (at least a pre-cursor for later editing by hand) | source code |

|  |  |
| --- | --- |
| outputCFG(self, cfg\_filename) | source code |

|  |  |
| --- | --- |
| prepVarNames(self, extvars, intvars) | source code |

|  |  |
| --- | --- |
| prepDEpars(self) | source code |

|  |  |
| --- | --- |
| prepAuxFns(self, auxfndict, pardict, makeMfiles=True)  Prepare auxiliary functions, and create MATLAB m-files for those corresponding to 'tau' and 'inf' functions, unless optional makeMfiles==False. | source code |

|  |  |
| --- | --- |
| dumpTrajData(self, traj, dt, filename, precise=True)  If precise=True (default), this trajectory dump to file may take several minutes: Uses the 'precise' trajectory sample option for trajectories with variable time-steps. | source code |

**Inherited from `object`**:
`__delattr__`,
`__getattribute__`,
`__hash__`,
`__new__`,
`__reduce__`,
`__reduce_ex__`,
`__repr__`,
`__setattr__`,
`__str__`


|  |  |
| --- | --- |
| Properties | [hide private] |

**Inherited from `object`**:
`__class__`


|  |  |
| --- | --- |
| Method Details | [hide private] |

|  |  |
| --- | --- |
| \_\_init\_\_(self, name=`'``'`, infty\_val=10000000000.0)  *(Constructor)* | source code |

The optional 'infty\_val' argument sets the substitute default finite
value used to represent 'infinity', for variables that are not given
explicit bounds.

Overrides: object.\_\_init\_\_

|  |  |
| --- | --- |
| dumpTrajData(self, traj, dt, filename, precise=True) | source code |

If precise=True (default), this trajectory dump to file may take
several minutes: Uses the 'precise' trajectory sample option for
trajectories with variable time-steps. Use precise=False option for
trajectories calculated at fixed time steps.

  


| Home | Trees | Indices | Help | PyDSTool |
| --- | --- | --- | --- | --- |

|  |  |
| --- | --- |
| Generated by Epydoc 3.0.1 on Fri May 4 15:24:08 2012 | http://epydoc.sourceforge.net |
